# Baicalein ameliorates osteoporosis via AKT/FOXO1 signaling

**DOI:** 10.18632/aging.203227

**Published:** 2021-07-01

**Authors:** Pan Cai, Yan Lu, Zhifeng Yin, Xiuhui Wang, Xiaoxiao Zhou, Zhuokai Li

**Affiliations:** 1 Department of Orthopedics, Shanghai University of Medicine and Health Sciences Affiliated Zhoupu Hospital, Shanghai, China; 2 Department of Laboratory Medicine, Shanghai University of Medicine and Health Sciences Affiliated Zhoupu Hospital, Shanghai, China; 3 Department of Orthopedics, Shanghai Zhongye Hospital, Shanghai 200941, China

**Keywords:** baicalein, osteoporosis, KEGG, AKT

## Abstract

In this study, we used bioinformatics and an *in vitro* cellular model of glucocorticoid-induced osteoporosis to investigate mechanisms underlying the beneficial effects of baicalein (BN) against osteoporosis. STITCH database analysis revealed 30 BN-targeted genes, including *AKT1, CCND1, MTOR*, and *PTEN*. Functional enrichment analysis demonstrated that BN-targeted genes were enriched in 49 Kyoto Encyclopedia of Genes and Genomes (KEGG) pathways. MIRWALK2.0 database analysis identified 110 enriched KEGG pathways related to osteoporosis. A Venn diagram demonstrated that 26 KEGG pathways were common between osteoporosis and BN-targeted genes. The top 5 common KEGG pathways were prostate cancer, bladder cancer, glioma, pathways in cancer, and melanoma. BN-targeted genes in the top 5 shared KEGG pathways were involved in PI3K-AKT, MAPK, p53, ErbB, and mTOR signaling pathways. In addition, glucocorticoid-induced osteoporosis in MC3T3-E1 cells was partially reversed by BN through inhibition of AKT, which, by upregulating FOXO1, enhanced expression of bone turnover markers (ALP, OCN, Runx2, and Col 1) and extracellular matrix mineralization. These findings demonstrate that BN suppresses osteoporosis via an AKT/FOXO1 signaling pathway.

## INTRODUCTION

Osteoporosis is a metabolic bone disease commonly diagnosed in the elderly [1]. Chronic osteoporosis is manifest by progressive brittleness of bones and greater incidence of non-stress fractures because of excessive bone resorption, low bone mineral density, and deterioration of bone micro-structure [2, 3]. Several genetic and environmental factors, such as Vitamin D deficiency and low estrogen levels, contribute to the progression of osteoporosis [4]. However, molecular mechanisms underlying osteoporosis remain unclear.

Baicalein (BN) is one of the most abundant flavonoid in *Scutellaria baicalensis*, which is widely used in Chinese herbal medicine for various ailments [5]. For example, BN reduces cerebrovascular resistance, improves cerebral blood circulation, and prevents platelet agglutination [5]. BN is clinically used for treatment of paralysis in patients with cerebrovascular disease [6]. The beneficial effects of BN on osteoporosis have also been reported. For example, BN acts as a lipoxygenase inhibitor and increases bone formation in osteoporosis model mice [7]. Moreover, BN promotes osteoblastic differentiation of MC3T3-E1 cells via protein kinases and transcription factors such as P-4E/BP1 and P-S6K1 [8]. However, the molecular mechanisms underlying the therapeutic effects of BN on osteoporosis are not clear.

Bioinformatics analysis has been widely used to unravel molecular and cellular mechanisms of several human diseases [9–11]. Therefore, in this study, we performed comprehensive bioinformatics analysis and *in vitro* experiments in the cellular model of glucocorticoid-induced osteoporosis (GIO) to identify molecular mechanisms underlying the therapeutic effects of BN in osteoporosis.

## RESULTS

### BN-targeted genes and the interaction network

**Figure 1:**
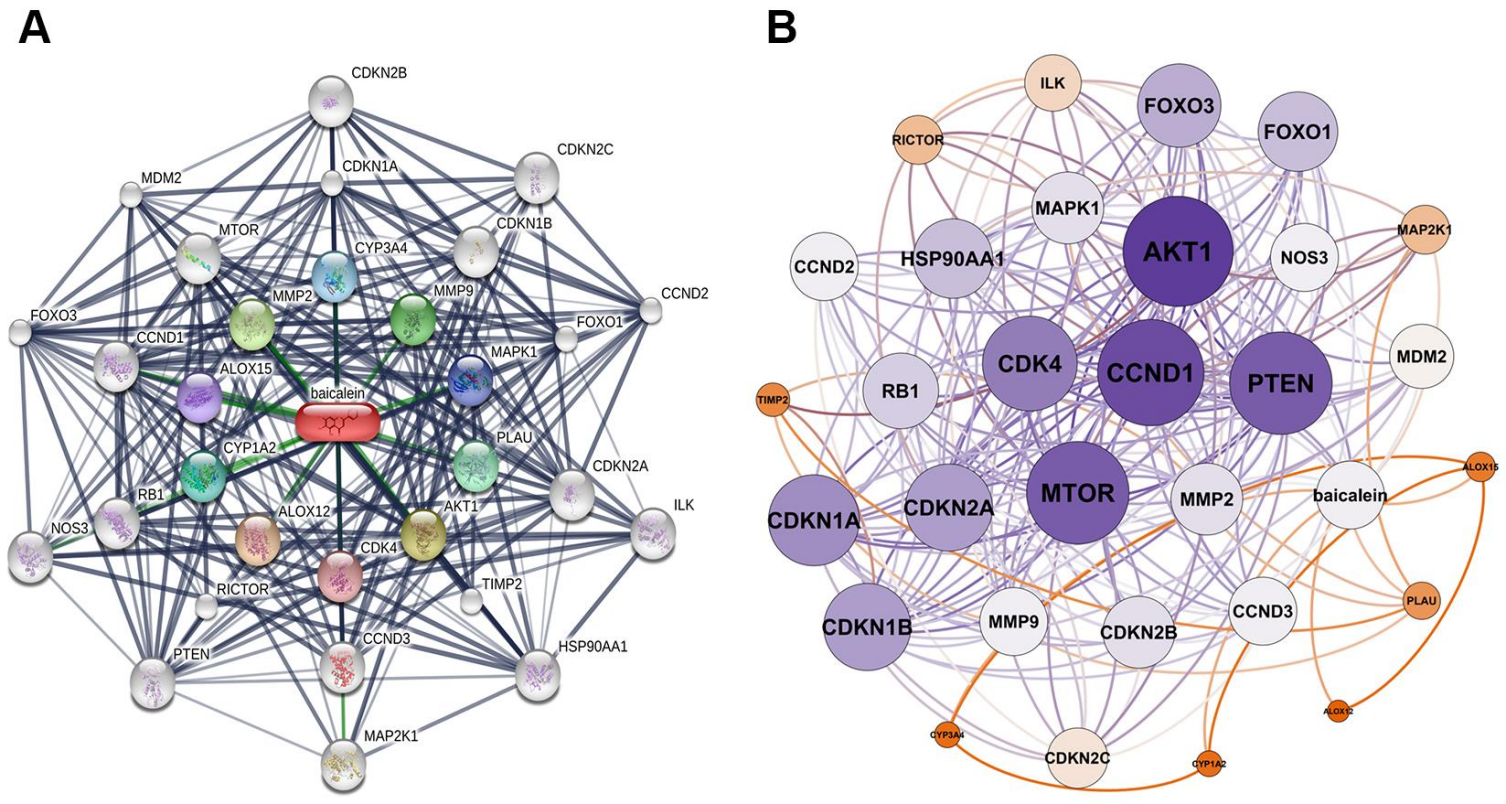
**Identification of baicalein-targeted genes.** (**A**) Identification of 30 baicalein-targeted genes using STITCH database. (**B**) Weighted interaction network analysis of baicalein-targeted genes using the STITCH database. The weights of *AKT1, CCND1, MTOR*, and *PTEN* were highest among BN-targeted genes.

We identified 30 baicalein-targeted genes belonging to three shells based on STITCH database analysis. The interaction network between these 30 baicalein-targeted genes is shown in Figure 1A. The first shell contained genes such as *CYP1A2, CYP3A4, CDK4, MMP2, AKT1, MMP9, MAPK1, PLAU, ALOX12,* and *ALOX15* and were probably the direct targets of baicalein. The second shell included genes such as *MTOR, RICTOR, TIMP2, RB1, CDKN1A, CDKN1B, CDKN2A, CCND3, FOXO1*, and *CCND1*. The third shell included genes such as *MAP2K1, NOS3, ILK, HSP90AA1, PTEN, FOXO3, MDM2, CDKN2C, CCND2*, and *CDKN2B*. The network of baicalein-targeted genes based on interaction weights is shown in Figure 1B. *AKT1, CCND1, MTOR*, and *PTEN* showed the highest interaction weights among the 30 baicalein-targeted genes.

### PPI network of BN-related genes

**Figure 2:**
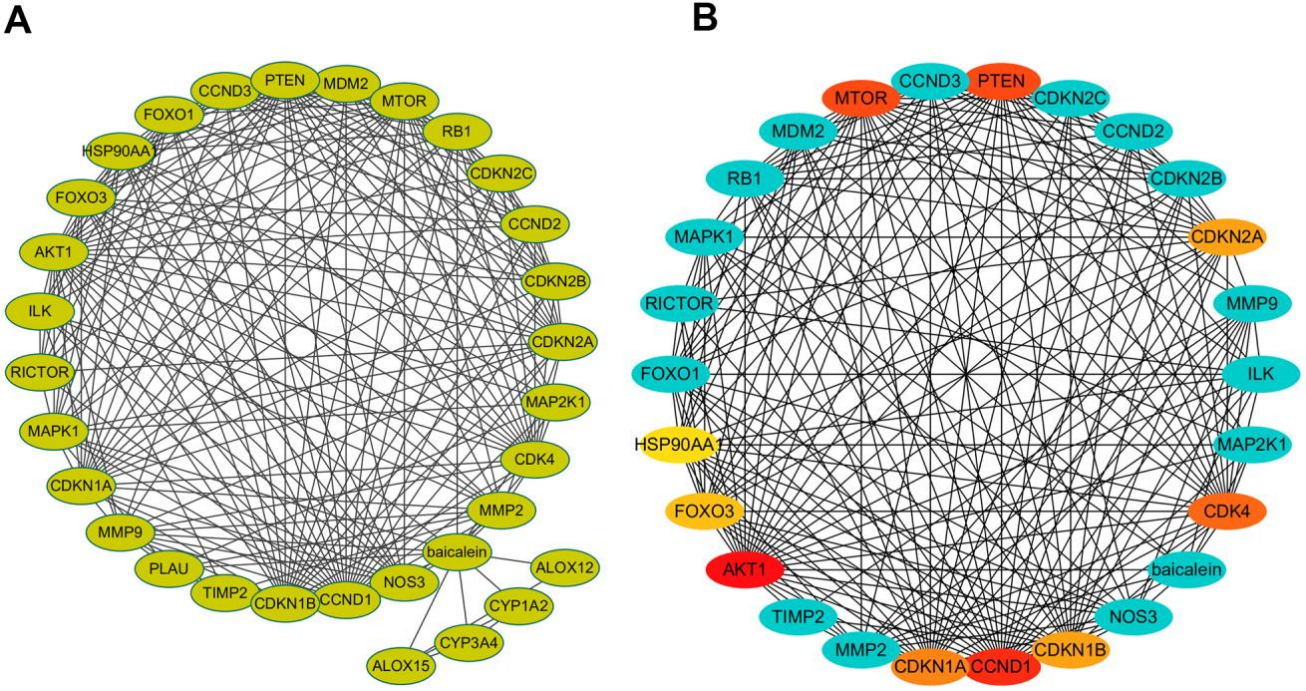
**PPI network of baicalein-targeted genes.** (**A**) PPI network of baicalein-targeted genes constructed using Cytoscape. (**B**) A list of baicalein-targeted genes in the PPI network ranked by degree connectivity. As shown, the top ten baicalein-targeted genes by degree connectivity were AKT1, CCND1, PTEN, MTOR, CDK4, CDKN1A, CDKN1B, CDKN2A, FOXO3, and FOXO1.

The protein-protein interaction (PPI) network of BN-related genes using Cytoscape (Figure 2A). The baicalein-targeted genes were ranked according to degree values (Figure 2B). The top ten genes based on degree values were *AKT1, CCND1, PTEN, MTOR, CDK4, CDKN1A, CDKN1B, CDKN2A, FOXO3*, and *FOXO1*.

### Identification of shared KEGG pathways between BN-targeted genes and osteoporosis

**Figure 3:**
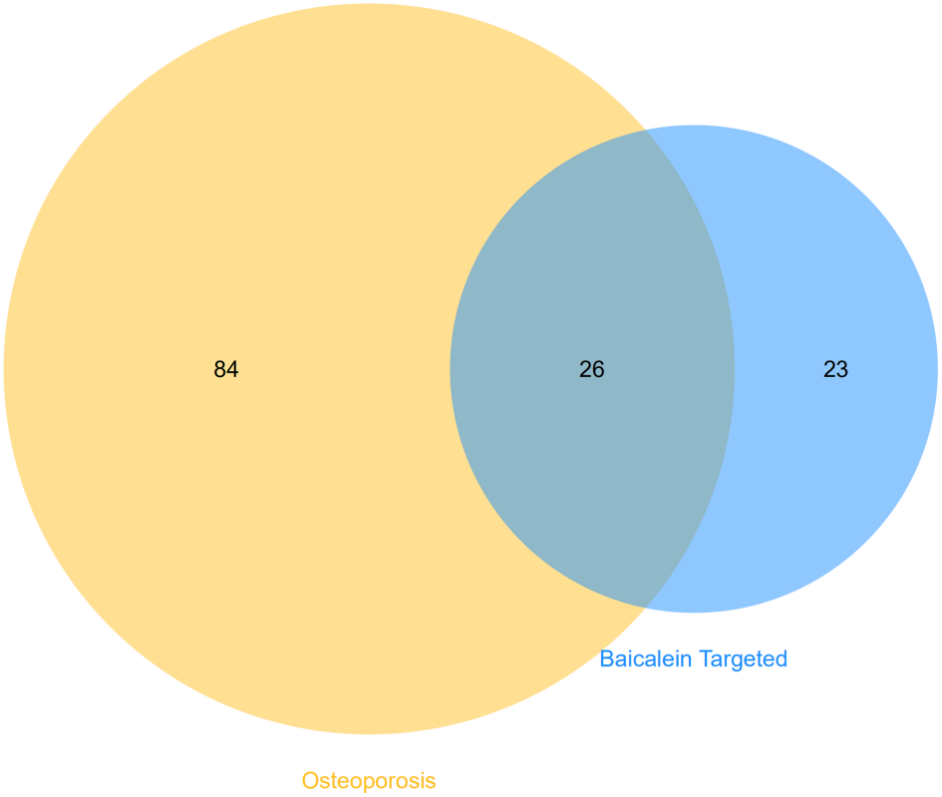
**Identification of shared KEGG pathways related to baicalein-targeted genes and osteoporosis.** Venn diagram shows 26 common KEGG pathways by intersecting those related to baicalein-target genes (n=49 KEGG pathways) and osteoporosis (n=110 KEGG pathways).

We identified 49 enriched KEGG pathways (p <0.05) by performing functional enrichment analysis of baicalein-targeted genes using DAVID. Furthermore, we identified 110 enriched KEGG pathways associated with human osteoporosis using the miRWalk2.0 database. We further identified 26 common KEGG pathways between osteoporosis and baicalein-targeted genes using the Venn diagram (Figure 3). Among these, prostate cancer, bladder cancer, glioma, pathway in cancer, and melanoma were the top five common KEGG pathways (Table 1).

**Table 1:** Top five KEGG pathway and involved genes.

**Term**	**KEGG pathway**	**Baicalein-target genes**	***Adj P*-value**
hsa05215	Prostate cancer	RB1, CDKN1A, MAP2K1, HSP90AA1, CDKN1B, CCND1, PTEN, MDM2, MAPK1, AKT1, FOXO1, MTOR	8.67E-13
hsa05219	Bladder cancer	RB1, CDKN1A, MAP2K1, CCND1, CDKN2A, CDK4, MMP2, MDM2, MAPK1, MMP9	8.67E-13
Hsa05214	Glioma	RB1, CDKN1A, MAP2K1, CCND1, CDKN2A, CDK4, PTEN, MDM2, MAPK1, AKT1, MTOR	8.67E-13
hsa05200	Pathways in cancer	RB1, CDKN1A, MAP2K1, CDKN2B, HSP90AA1, CDKN1B, CDKN2A, MMP2, PTEN, MMP9, FOXO1, MTOR, CCND1, CDK4, MDM2, AKT1, MAPK1	3.17E-12
Hsa05128	Melanoma	RB1, CDKN1A, MAP2K1, CCND1, CDKN2A, CDK4, PTEN, MDM2, MAPK1, AKT1	6.22E-11

### Identification of baicalein-targeted hub genes

**Figure 4:**
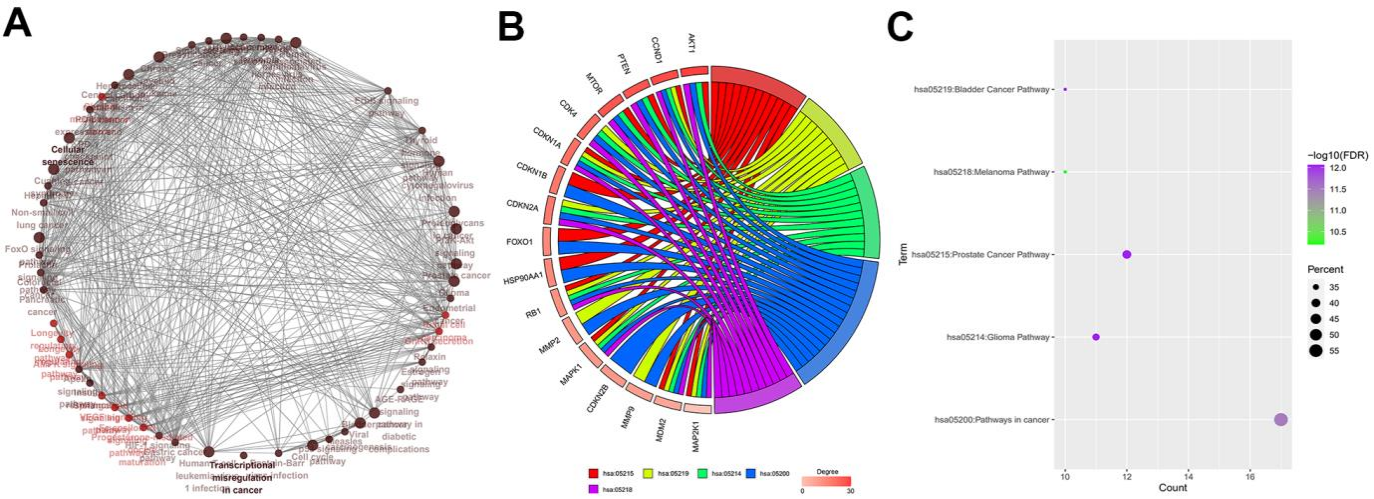
**Identification of hub genes.** (**A**) Relationship and interactions between baicalein-targeted genes in the top 5 enriched KEGG pathways. (**B**) Functional enrichment analysis results of baicalein-targeted genes. *CCND1, CDKN1A, RB1, MAPK1, MDM2*, and *MAP2K1* were common to all the top five shared KEGG pathways and were designated as hub genes. The top five genes based on degree connectivity were *AKT1, CCND1, PTEN, MTOR*, and *CDK4*. (**C**) The FDR values, gene numbers, and rich factor values (ratio of the number of enriched DEGs in the KEGG pathway category compared to the total number of genes in that category) of the top five shared KEGG pathways.

Functional enrichment analysis of BN-targeted genes is shown in Figure 4A. Among the 30 BN-targeted genes, *AKT1, CCND1, PTEN, MTOR, CDK4, CDKN1A, CDKN1B, CDKN2A, FOXO1, HSP90AA1, RB1, MMP2, MAPK1, CDKN2B, MMP9, MDM2*, and *MAP2K1* were involved in the top five KEGG pathways. Furthermore, we identified *CCND1, CDKN1A, RB1, MAPK1, MDM2,* and *MAP2K1* were identified as hub genes (Figure 4B). The FDR values, gene counts, and rich factors are shown in Figure 4C. The KEGG pathway, hsa05200: pathways in cancer, showed the highest FDR value, rich factor, and gene numbers (Figure 4C).

### Analysis of KEGG pathways related to BN-targeted genes

**Figure 5:**
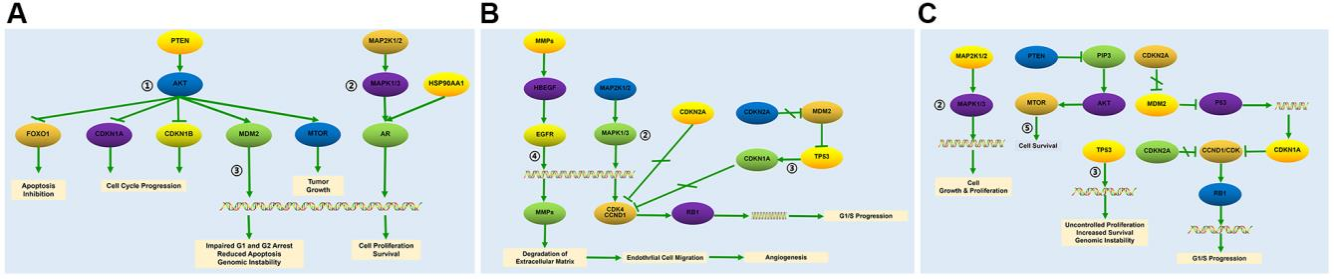
**Baicalein-targeted genes in the top three KEGG pathways.** The list of baicalein-targeted genes among (**A**) PI3K-AKT, MAPK, and p53 signaling pathway genes enriched in prostate cancer; (**B**) MAPK, p53, and ErbB signaling pathway genes enriched in bladder cancer, and (**C**) MAPK, p53, and mTOR signaling pathway genes enriched in pathways in cancer.

The top 5 baicalein-targeted KEGG pathways were associated with apoptosis inhibition, cell cycle progression, impaired G1 and G2 cell cycle arrest, genomic instability, tumor growth, cell growth and proliferation, angiogenesis, G1/S cell cycle progression, uncontrolled proliferation, and increased survival (Figure 5). This suggested that baicalein regulated osteogenesis through PI3K-AKT, MAPK, p53, ErbB, and mTOR signaling pathways.

### BN partially reverses GIO through suppression of AKT expression

**Figure 6:**
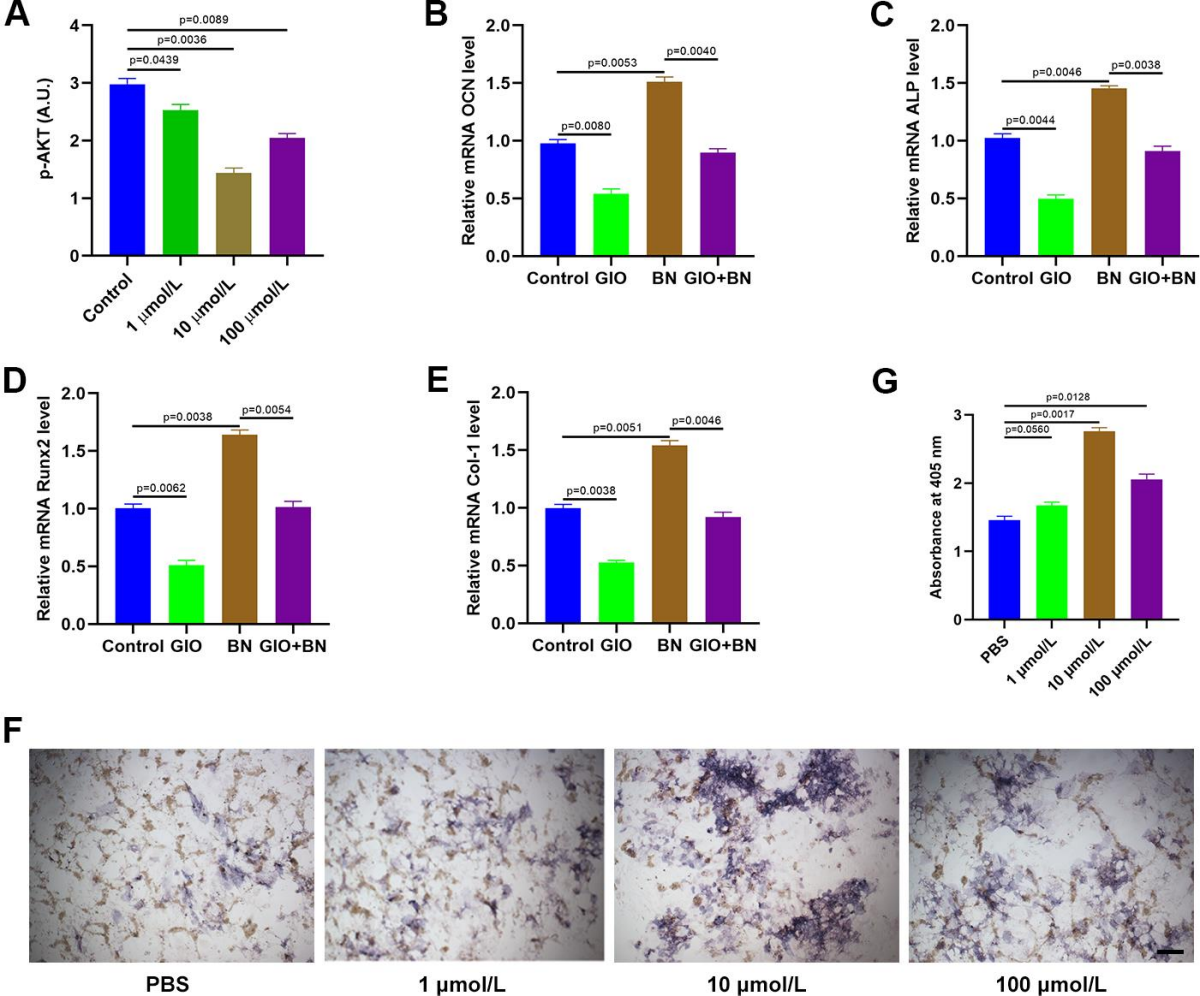
**BN treatment partially reverses GIO by suppressing AKT.** (**A**) ELISA analysis shows phospho-AKT levels in GIO-induced MC3T3-E1 cells treated with BN (1μM, 10 μM, and 100 μM). (**B**–**E**) QRT-PCR analysis shows expression levels of bone turnover markers (ALP, OCN, Runx2, and Col 1) in GIO model MC3T3-E1 cells treated with BN (10 μM, 10 μM, and 100 μM BN). (**F**, **G**) ALP staining results of GIO model MC3T3-E1 cells treated with BN (1μM, 10 μM, and 100 μM). The data are represented as means±SD of at least 3 independent experiments.

We then analyzed AKT phosphorylation levels in MC3T3-E1 cells treated with different concentrations of baicalein (1 μM, 10 μM, and 100 μM) by ELISA. BN treatment significantly reduced p-AKT levels in MC3T3-E1 cells; p-AKT levels were significantly lower in the 10 μM BN-treatment group compared to the 1 μM and 100 μM BN-treatment groups (Figure 6A). QRT-PCR analysis results showed that the GIO treatment significantly decreased the expression levels of bone turnover markers such as ALP, OCN, Runx2, and Col 1 in MC3T3-E1 cells, but these effects were partially reversed by BN (Figure 6B–6E). Furthermore, ALP staining assay results showed that BN partially rescued impaired extracellular matrix mineralization in GIO-treated MC3T3-E1 cells (Figure 6F, 6G).

### BN regulates osteogenesis via AKT/FOXO1 signaling

**Figure 7:**
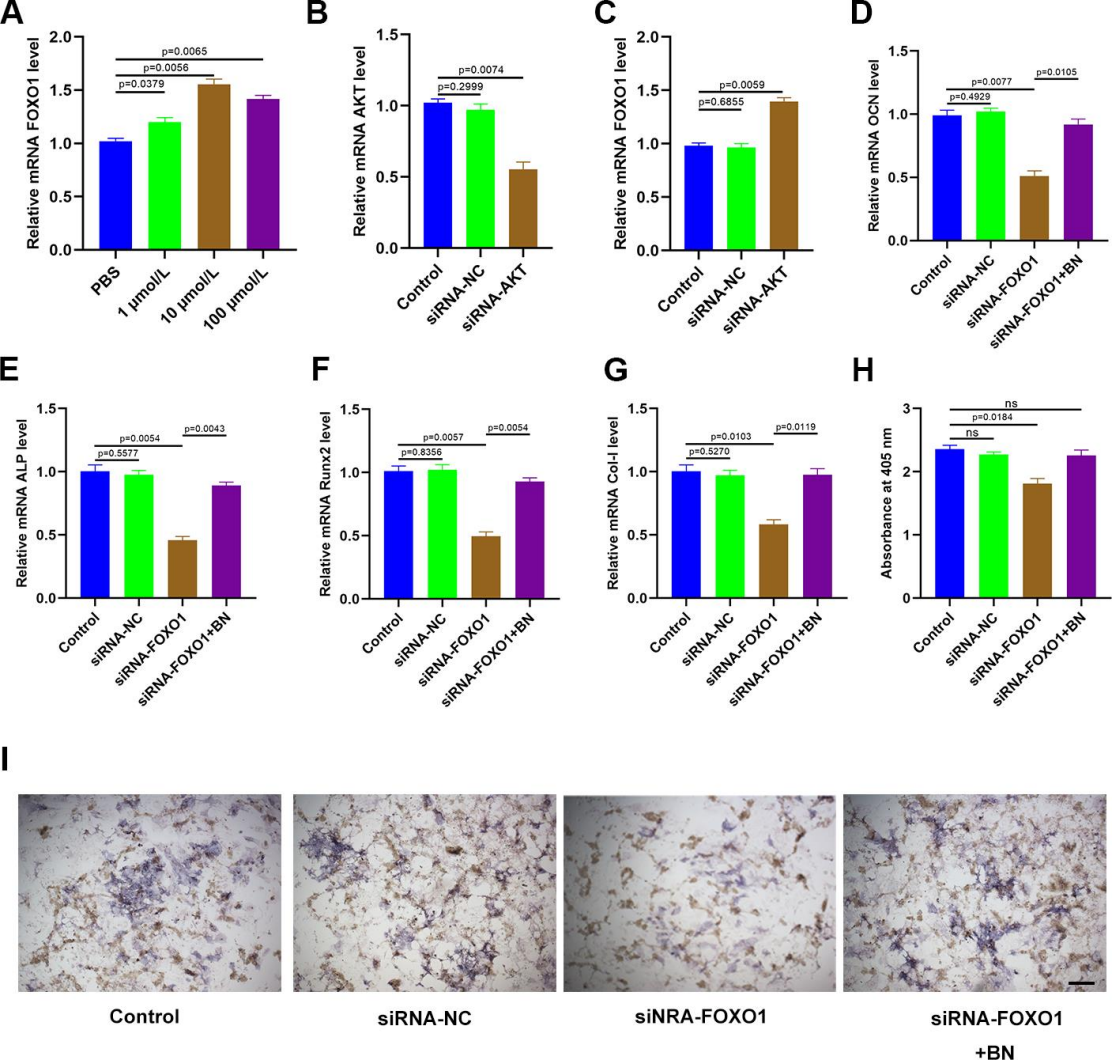
**BN regulates osteogenesis via AKT/FOXO1 signaling.** (**A**) QRT-PCR analysis shows FOXO1 levels in GIO model MC3T3-E1 cells treated with BN (1μM, 10 μM, and 100 μM). (**B**) QRT-PCR analysis shows AKT mRNA levels in control-siRNA- and AKT siRNA-transfected MC3T3-E1 cells. (**C**) QRT-PCR analysis shows the levels of FOXO1 in control-siRNA- and AKT siRNA-transfected GIO model MC3T3-E1 cells treated with BN (1μM, 10 μM, and 100 μM). (**D**–**G**) QRT-PCR analysis shows the levels of bone turnover markers (ALP, OCN, Runx2, and Col 1) in control-siRNA- and AKT siRNA-transfected GIO model MC3T3-E1 cells treated with BN (1μM, 10 μM, and 100 μM). (**H**, **I**) ALP staining results of control-siRNA- and AKT siRNA-transfected GIO model MC3T3-E1 cells treated with BN (1μM, 10 μM, and 100 μM). The data are shown as means±SD of 3 independent experiments.

QRT-PCR analysis showed that expression of FOXO1 was significantly increased in BN-treated MC3T3-E1 cells; FOXO1 levels were highest in the 10 μM BN-treatment group (Figure 7A). Furthermore, qRT-PCR analysis confirmed that AKT levels were significantly reduced in AKT-siRNA-transfected MC3T3-E1 cells compared to the corresponding controls (Figure 7B). AKT silencing significantly increased FOXO1 levels in MC3T3-E1 cells (Figure 7C). QRT-PCR analysis showed that FOXO1 silencing significantly reduced expression levels of the bone turnover markers (ALP, OCN, Runx2, and Col 1) in GIO-treated MC3T3-E1 cells, but these effects were partially reversed by BN treatment (Figure 7D–7G). ALP staining results showed that BN treatment partially rescued impaired extracellular matrix mineralization in GIO-treated FOXO1-silenced MC3T3-E1 cells (Figure 7H, 7I).

## DISCUSSION

Osteoporosis is a chronic metabolic bone disorder observed commonly in aged individuals, and is characterized by low bone mineral density, increased brittleness of bone, damaged bone micro-structure, and significant increase in the number of non-stress fractures [12]. BN is one of the most abundant flavonoids in *Scutellaria baicalensis* and is widely used in Chinese herbal medicine to treat various human diseases for centuries. [13]. BN promotes osteogenic differentiation and bone formation [14–16], but the molecular mechanisms underlying the beneficial effects of BN on osteoporosis are not clear. Pathogenetic mechanisms underlying several human diseases have been discovered through bioinformatics analyses of microarray data and whole-genome sequencing studies [17].

In the present study, we identified 110 KEGG pathways that were associated with differentially expressed genes (DEGs) in human osteoporosis and 49 KEGG pathways associated with BN-targeted genes. PI3K-AKT, MAPK, p53, ErbB, and mTOR signaling pathways were the top five shared KEGG pathways among these two groups.

Previous studies have shown that BN-targeted genes in the PI3K-AKT signaling pathway were associated with osteogenesis [18, 19]. Many studies have shown that PI3K-AKT signaling pathway plays a significant role in osteoporosis [20, 21]. For an example, a recent study demonstrated that inhibition of PI3K-AKT signaling pathway delayed osteoporosis progression in postmenopausal women by suppressing inflammation and formation of osteoclasts [22]. Xiao et al demonstrated that inhibition of miR-148a ameliorated ovariectomy-induced osteoporosis via PI3K-AKT signaling pathway [23]. In the present study, we showed that BN significantly suppressed AKT expression and activation in MC3T3-E1 cells. BN treatment also stimulated the expression of bone turnover markers and enhanced extracellular matrix mineralization in GIO-induced MC3T3-E1 cells. These results suggested that the BN treatment suppresses osteoporosis via AKT.

FOXO family of transcription factors such as FOXO1, FOXO3, FOXO4, and FOXO6 play a crucial role in cellular defense mechanisms against oxidative stress [24]. Liao et al reported that FOXO1 knockdown decreased osteoblast differentiation by suppressing the expression of antioxidant enzymes, manganese superoxide dismutase and catalase, thereby increasing the levels of reactive oxygen species [25]. Feng et al demonstrated that inhibition of PI3K-AKT signaling pathway significantly increased FOXO1 transcriptional activity, which suppressed osteoporosis by enhancing antioxidant mechanisms [26]. In our study, we demonstrated that BN promoted expression of FOXO1 by suppressing AKT. We also observed that BN treatment and AKT silencing improved osteogenic differentiation of GIO-induced MC3T3-E1 cells by increasing FOXO1 expression. Therefore, our results demonstrate that BN treatment suppresses osteoporosis via AKT/FOXO1 signaling pathway. Future studies are required to confirm our findings *in vivo* and further investigate mechanisms underlying the beneficial effects of BN on osteogenesis.

## MATERIALS AND METHODS

### Cell culture and transfection

The murine pre-osteoblast cell line, MC3T3-E1, was a kind donation from the Shanghai University of Medicine and Health Sciences (Shanghai, China). MC3T3-E1 cells were grown in α-MEM medium (SH30265.01B; Hyclone) containing 10% fetal bovine serum (FBS; Gibco) and 1% penicillin and streptomycin at 37° C, 5% CO_2_, and 95% humidity. MC3T3-E1 cells up to five passages were used for experiments. The cells were transfected with 50 nM AKT siRNA, 50 nM FOXO1 siRNA and their corresponding control siRNAs using lipofectamine 3000. MC3T3-E1 cells were treated with 100 μM dexamethasone (CAS: 50-02-2, purity: >98%; ACMEC, Shanghai, China) for 7 days to induce the cellular GIO model. The cells were treated with 1 μM, 10 μM, and 100 μM baicalein (Cat. No. HY-N0196-100 mg; MedChemExpress) for various time points as indicated.

### Quantitative real-time PCR (qRT-PCR)

Total RNA was extracted using TRIzol according to manufacturer’s instructions. Then, cDNA synthesis was performed using the one-step Prime Script miRNA cDNA synthesis kit. Then, qPCR analysis was performed using SYBR Premix Ex TaqII (TaKaRa, Japan) in the Thermal Cycler C-1000 Touch system (CFX Manager, 10021377; Bio-Rad, USA). The expression levels of various genes were determined using the 2^-ΔΔCt^ method and normalized to GAPDH. The primers used for qPCR analysis were as follows: AKT-forward, 5′- ATGAGCGACGTGGCTATTGT- 3′, AKT-reverse, 5′- GAGGCCGTCAGCCACAGTCT- 3′; FOXO1-forward, 5′- AGGGTTAGTGAGCAGGTTACAC- 3′,

FOXO1-reverse, 5′- TGCTGCCAAGTCTGACGAAA- 3′;

ALP-forward, 5′- TGACTACCACTCGGGTGAACC- 3′,

ALP-reverse, 5′- TGATATGCGATGTCCTTGCAG- 3′;

OCN-forward, 5′- TTCTGCTCACTCTGCTGACCC- 3′,

OCN-reverse, 5′- CTGATAGCTCGTCACAAGCAGG- 3′;

Runx2-forward, 5′- CGCCACCACTCACTACCACAC- 3′,

Runx2-reverse,5′- TGGATTTAATAGCGTGCTGCC- 3′;

Col-1-forward, 5′- AACTTTGCTTCCCAGATGTCC- 3′,

Col-1-reverse, 5′- AGCCTCGGTGTCCCTTCA- 3′;

GAPDH-forward, 5′- GAAGGTCGGTGTGAACGGATTTG- 3′,

GAPDH-reverse, 5′- CATGTAGACCATGTAGTTGAGGTCA- 3′.

### ELISA

MC3T3-E1 cells were grown for 48 h in serum-free medium. The concentration of phospho-AKT in each group of cells was measured using the ELISA kit according to manufacturer’s instructions. The colorimetric values were normalized to total cell numbers in each well. Then, the phospho-AKT levels in each sample were calculated using the standard curve.

### ALP staining

MC3T3-E1 cells were washed twice with PBS and then fixed with 10% formalin for 15 minutes. Then, the cells were processed using the ALP color-development kit according to manufacturer’s instructions and the color development was performed by incubating cells with BCIP/NBT liquid substrate for 24 hours. Absorbance was measured at 405 nm in a plate reader. The experiments were performed in triplicate.

### Identification of BN-target genes and construction of PPI network

We identified baicalein-targeted genes and constructed the protein-protein interaction (PPI) network between them using the Search Tool for Interacting Chemicals (STITCH) database [27]. We analyzed the PPI network of baicalein-targeted genes using the Cytoscape 3.7.2 version software [28] and measured the degree, betweenness, and closeness of each gene in the network. The hub genes were identified based on the degree analysis using the cytoHubba plugin. Cytoscape plug-in ClueGO was used to identify enriched KEGG pathways representing the baicalein-targeted genes and the relationship between them. The KEGG pathway enrichment results were visualized using the ggplot2.7 package.

### Identification of shared KEGG pathways between BN-targeted genes and osteoporosis

MiRWalk2.0 software [29] was used to retrieve human osteoporosis-related KEGG pathways using p<0.05 as the threshold parameter. VennDiagram (http://www.ehbio.com/ImageGP/index.php/Home/Index/index.html) was then used to identify shared KEGG pathways between human osteoporosis and baicalein-targeted genes.

### Identification of hub genes

GOplot R package [30] was used to visualize functional enrichment analysis of KEGG pathways enriched with baicalein-targeted genes and identified hub genes from among the top five shared KEGG pathways between baicalein-targeted genes and osteoporosis.

### Identification of KEGG pathways related to BN-targeted genes

We selected the top 5 shared KEGG pathways with the smallest p values and used ScienceSlides to schematically represent KEGG pathways related to baicalein-targeted genes, the hub genes and their related mechanisms of action.

### Statistical analysis

The data are represented as means ± SD. Prism 8.0 software was used for all statistical analyses. One-way analysis of variance with Tukey’s post hoc test was used to compare three or more groups of data. Two-tailed Student’s test was used to compare data between two groups. P < 0.05 was considered statistically significant.
